# Synergistic effects of zeolite imidazole framework@graphene oxide composites in humidified mixed matrix membranes on CO_2_ separation

**DOI:** 10.1039/c7ra09794h

**Published:** 2018-02-07

**Authors:** Dandan Huang, Qingping Xin, Yazhou Ni, Yingqian Shuai, Shaofei Wang, Yifan Li, Hui Ye, Ligang Lin, Xiaoli Ding, Yuzhong Zhang

**Affiliations:** State Key Laboratory of Separation Membranes and Membrane Processes, School of Materials Science and Engineering, Tianjin Polytechnic University Tianjin 300387 P. R. China xinqingping@tjpu.edu.cn zhangyz2004cn@vip.163.com; School of Chemical Engineering and Energy, Zhengzhou University Zhengzhou 450001 P. R. China

## Abstract

In this study, composite nanosheets (ZIF-8@GO) were prepared *via* an *in situ* growth method and then incorporated into a polyimide (PI) matrix to fabricate mixed matrix membranes (MMMs) for CO_2_ separation. The as-prepared MMMs were characterized by Fourier transform infrared (FT-IR) spectroscopy, scanning electron microscopy (SEM), X-ray diffraction (XRD), differential scanning calorimetry (DSC), thermogravimetric analyses (TGA) and water uptake measurements. Water uptake measurements establish the relationship between the gas permeability and water uptake of membranes and an increase in the water uptake contributes to the CO_2_ permeability owing to an increase in the CO_2_ transport channels. The MMMs exhibit excellent CO_2_ permeability in when compared with an unfilled PI membrane in a humidified state. The ZIF-8@GO filled membranes can separate CO_2_ efficiently due to the ZIF-8@GO nanocomposite materials combining the favorable attributes of GO and ZIF-8. First, the high-aspect ratio of the GO nanosheets enhances the diffusivity selectivity. Second, ZIF-8 with a high surface area and microporous structure is beneficial to the improvement of the CO_2_ permeability. Third, ZIF-8@GO possesses synergistic effects for efficient CO_2_ separation. The MMM with 20 wt% ZIF-8@GO exhibits the optimum gas separation performance with a CO_2_ permeability of 238 barrer, CO_2_/N_2_ selectivity of 65, thus surpassing the 2008 Robeson upper bound line.

## Abstract 2

In this study, composite nanosheets (ZIF-8@GO) were prepared *via* an *in situ* growth method and then incorporated into a polyimide (PI) matrix to fabricate mixed matrix membranes (MMMs) for CO_2_ separation.

## Graphical abstract

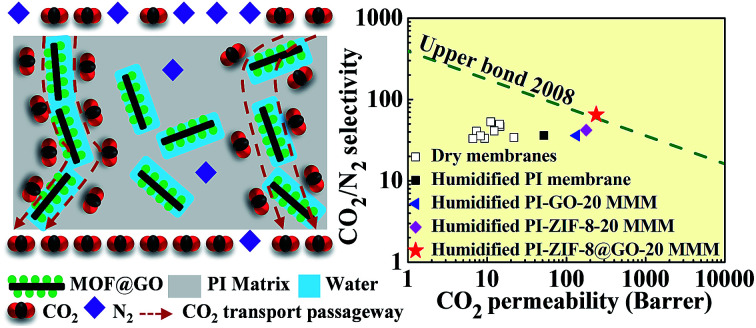

## 1 Introduction

CO_2_, as the main greenhouse gas, has received extensive attention in order to reduce its emission all over the world. Membrane separation, as an attractive alternative of conventional techniques, has developed rapidly due to its high efficiency, low cost, and energy saving and environment-friendly characteristics and has become one of the promising CO_2_ separation technologies in the field of carbon capture technology.^1–4^ Polymer membrane materials have good processing performance; however, their gas separation performance suffers from trade-off properties. Inorganic membranes have excellent separation performance, but they are difficult to process. Mixed matrix membranes (MMMs) with organic and inorganic materials embedded into polymeric membranes overcome this trade-off limit and realize the simultaneous improvement of selectivity and permeability.^5,6,7^ Different from the pure polymer, the MMM has multiple functions, multi-level structure, multiple phases and multiple functions, which provide a wealth of possibilities for the design and preparation of MMMs, thus becoming a hot spot in recent years. However, due to the differences in the physical and chemical characteristics of the polymer and inorganic filler, poor interface morphologies such as interface defects and interface cavities could be easily produced. Therefore, to create a good interface morphology between the polymer and filler is key to preparing high-performance MMMs. Such filler-materials include zeolites,^8^ carbon molecular sieves (CMS),^9^ metal-based oxides,^10–13^ silica,^14,15^ carbon nanotubes,^16,17^ graphene oxide,^18,19^ metal organic frameworks,^20,21^ and covalent organic frameworks.^22,23^

Due to their high surface area and porous properties, metal organic frameworks (MOFs) are widely used as membranes in gas separation processes.^24,25^ MOF membranes have been researched for their gas separation performances; these membranes often show high gas separation performances because of their rigid pores and uniformity. However, ultra-thin MOF membrane fabrication has a significant challenge that MOF membranes usually need to be supported because they do not have enough mechanical strength to support themselves. Moreover, their high cost and complex manufacturing and processing have limited their widespread industrial applications.

An alternative approach is to embed the porous MOF materials into a polymer matrix to fabricate MMMs. MMMs may combine the advantages of both the filler phase with uniform pores and the polymer phase with superior mechanical strength and easy fabrication.^26^ The favorable properties of the two phases are endowed in the MMMs and overcome the defects of a single material, generating additional synergy. The functional filler plays a key role in the membrane structure because its pore size distribution determines the separation performance.^27^ In other words, fillers with a well-defined pore size and shape increase the porosity of the MMMs and provide more gas permeation and diffusion channels. Vankelecom *et al.* fabricated MMMs by incorporating Cu_3_(BTC)_2_ into the polymer matrix and found that the CO_2_ permeability of the PI/30 wt% [Cu_3_(BTC)_2_] membrane was 80% higher than that of the unfilled membrane.^28^ Kaliaguine *et al.* fabricated CO_2_/CH_4_ gas separation MMMs and investigated the effect of modifying the MOF structure with –NH_2_ functional groups in CO_2_/CH_4_ gas separation.^29^ It was found that the MMMs loaded with MOF-199 increased both the CO_2_ permeability and ideal selectivity by 49% and 16%, respectively, while the MMMs loaded with NH_2_-MOF-199 increased by 82% and 35% both in CO_2_ permeability and ideal selectivity when compared with the unfilled membrane. MOF-5 containing MMMs were prepared by Musselman *et al.*^30^ and the permeability of gases was enhanced by 120%, while the CO_2_/CH_4_ selectivity increased by 6% at 30% MOF-5 loading. Gascon *et al.* incorporated 1,4-benzenedicarboxylate(CuBDC) MOF nanosheets into Matrimid® 5218 polymer to fabricate a MOF-polymer thin membrane.^20^ The ultrathin membrane shows outstanding CO_2_ separation performance from CO_2_/CH_4_ gas mixtures. Liu *et al.*^31^ reported the permeability of H_2_ and the H_2_/CO_2_ selectivity of 6 wt% Cu_3_(BTC)_2_ MMM increased by 45% and a factor of 2.78 when compared with pure PI. Subsequently, Hu *et al.*^32^ compared the effect of three types of fillers (MOF-5, Cu_3_(BTC)_2_, and MIL-53(Al)) on the gas separation performance and proved that the Cu_3_(BTC)_2_ loaded membrane had the best separation performance.

Graphene oxide (GO) as a well-known two-dimensional material possesses a unique one-atom-thick structure.^33^ These properties endowed GO to become a promising material for use in separation membranes. GO nanosheets can assemble a graphene laminate membrane and GO can be used as the filler embedded in a polymer matrix to obtain MMMs.^34,35^ GO-based membranes are predicted to be highly selective owing to their inherent 2D channels. The composites of MOF and GO, such as ZIF-8@GO^36^ and MOF-505@GO,^37^ have attracted great attention owing to their advantageous gas separation performances. The MOF@GO may develop new pores at the interface of the MOF and GO surfaces and the CO_2_ separation will be enhanced due to the new porosity. Recently, MOF@GO materials used as fillers to prepare MMMs have been reported. Dong *et al.* fabricated MMMs by incorporating ZIF-8@GO into a Pebax® matrix and investigated their CO_2_ separation performance.^38^ The membrane showed the CO_2_ permeability and CO_2_/N_2_ selectivity of MMMs was 249 barrer and 47.6, respectively at 6 wt% ZIF-8@GO loading. The MOF@GO loaded membranes have good compatibility at the filler/polymer interface owing to the presence of GO.^37^ Moreover, this type of membrane can combine the advantages of the two materials.

In this study, MOF@GO was prepared as a filler to fabricate MMMs to enhance the CO_2_ separation performance. ZIF-8 was selected as a multifunctional filler because of its uniform pore and high thermal and chemical stability. GO was selected as the support for ZIF-8 due to its high surface area and abundant surface functional groups. Matrimid® 5218 was used as the polymer matrix due to its superior chemical and thermal properties. The ZIF-8@GO composite nanosheets were used as fillers embedded into the polymer matrix to fabricate a series of MMMs and the CO_2_ separation performance of the MMMs was investigated. Moreover, the influence of the water uptake and pressure on the gas separation performance was studied. In addition, the microstructure and thermal properties of the MMMs were revealed.

## 2 Experimental

### 2.1 Materials

Polyimide (PI, Matrimid® 5218) was supplied by Huntsman Advanced Materials Americas Inc. Zn(NO_3_)_2_·6H_2_O and 2-methylimidazole were purchased from Aladdin. Potassium permanganate (KMnO_4_), sodium nitrate (NaNO_3_), hydrochloric acid (HCl), and concentrated sulfuric acid (H_2_SO_4_, 98 wt%) were obtained from Tianjin Jiangtian Ltd. (Tianjin, China). Methanol and hydrogen peroxide aqueous solution (H_2_O_2_, 30 wt%) and *N*,*N*-dimethyl acetamide (DMAc) were obtained from Kemiou Chemical Reagent Co., Ltd. (Tianjin, China). Deionized water was used throughout the experiments.

### 2.2 Preparation of ZIF@GO

ZIF-8 particles were synthesized according to a literature procedure.^34^ Zn(NO_3_)_2_·6H_2_O (98.0 wt%, 1.464 g) and 2-methylimidazole (Hmim, 99.0 wt%, 3.244 g) were dissolved in 48 mL and 80 mL of methanol under stirring, respectively, and then mixed. The mixed solution was stirred for 3 h at 30 °C. The products were collected by centrifugation and washed three times with methanol. Finally, the as-obtained ZIF-8 was dried under vacuum.

GO was prepared using the modified Hummers method.^40^ Natural graphite powder (2.0 g) and NaNO_3_ (1.0 g) were dissolved in concentrated H_2_SO_4_ (150 mL) under stirring in an ice bath. Then, KMnO_4_ (7.0 g) was added slowly to the mixture with stirring over 1 h, while the temperature was maintained at ∼5 °C. The mixture was stirred at 55 °C for 4 h. Then, 150 mL of ice-cold deionized water was added into the mixture and then, the mixture was heated to 97 °C and kept at this temperature for 30 min. Finally, 50 mL of deionized water and 30 mL of H_2_O_2_ were added to the mixture, in sequence, with stirring. The mixture was centrifuged at 6000 rpm for 15 min and washed three times with 300 mL of HCl aqueous solution. Then, the mixture was washed with water until the filtrate was neutral. The product was dispersed in a certain amount of water. An aqueous suspension of GO at a concentration of 5 mg mL^−1^ was obtained. Then, the GO suspension was further diluted to 1 mg mL^−1^ using methanol and sonicated for 8 h prior to use.

The ZIF-8@GO nanosheets were prepared *via* the same process used for the preparation of ZIF-8 along with the addition of 8 mL of the as-prepared GO suspension. To prepare the ZIF-8@GO sample, Zn(NO_3_)_2_·6H_2_O (0.366 g) and 2-methylimidazole (0.811 g) were dissolved in 12 mL and 20 mL of methanol, respectively, and then mixed to obtain a mixed solution under stirring. Immediately, 8 mL of the as-prepared GO suspension was added to the above mixed solution and stirred for 3 h. Then, the mixture was washed and centrifuged at least three times and the products were dried in a vacuum oven.

### 2.3 Preparation of MMMs

The PI-ZIF-8@GO loaded MMMs and unfilled PI membrane were fabricated using a solution casting method. The PI powder (0.6 g) was dissolved in DMAc (6 mL) under stirring for 1 h and a desired amount of ZIF-8 or ZIF-8@GO was homogeneously dispersed into another vial containing 6 mL of DMAc *via* ultrasonication for 2 h. Then, the suspension was mixed with the PI solution and stirred for 12 h. The mixed suspension was cast onto a glass slide, dried at 50 °C for 12 h and then at 80 °C for 12 h. The MMMs were designated as PI-ZIF-8@GO-*x*, where *x* is the weight percentage of the fillers relative to the PI matrix.

### 2.4 Characterization of filler and membranes

Size and morphology of the GO and ZIF-8@GO were observed by transmission electron microscopy (TEM Hitachi H7650). The FT-IR spectra of GO, ZIF-8, ZIF-8@GO and the MMMs were recorded on a BRUKER Vertex 70 FT-IR spectrometer over the range of 4000–400 cm^−1^. The morphology of ZIF-8 and the cross-sectional structure of the membranes were obtained by scanning electron microscopy (SEM, S-4800). The TGA of the membranes were conducted using a STA449F3 apparatus. The measurements were tested from 40 °C to 800 °C under N_2_ atmosphere. The glass transition temperature of the membranes was studied using a DSC200F3 apparatus over the temperature range of 200–400 °C under N_2_ atmosphere. The crystalline structure of the fillers and membranes were recorded on a D8 DISCOVER X-ray diffractometer (XRD) over the range of 5–40°.

The water uptake and water state of the membranes have an important influence on the gas transport mechanism and were studied using a literature procedure.^39^ The membranes were weighed (*m*_1_, mg) after the gas permeation test under humidified conditions. Then, the membranes were dried at 100 °C for 6 h to remove any free water and weighed again (*m*_2_, mg). Finally, the membranes were dried at 150 °C for 6 h to remove any bound water and their absolute dry weight (*m*_0_, mg) was measured. The content of total water (*W*_t_, %), free water (*W*_f_, %) and bound water (*W*_b_, %) were acquired using eqn (1)–(3), respectively.

1 *W*_t_ = (*m*_1_ − *m*_0_)/*m*_0_ × 100%

2 *W*_f_ = (*m*_1_ − *m*_2_)/*m*_0_ × 100%

3 *W*_b_ = (*m*_2_ − *m*_0_)/*m*_0_ × 100%

### 2.5 Gas permeation experiment

A single gas (CO_2_, N_2_) permeation test of the humidified membranes was conducted using the constant pressure/variable volume method at 35 °C. Before the gas separation test, all the membranes were soaked in water for over two weeks to absorb adequate amount of water. In the measurement process, both the feed gas and sweep gas were saturated with water vapor by a bubbling method at 35 °C and then passing through an empty bottle at room temperature to remove the condensed water. N_2_ was used as the sweep gas for CO_2_, otherwise CO_2_ was used as the sweep gas for N_2_. The gas permeability (*P_i_*, barrer, 1 barrer = 10^−10^ cm^3^ (STP) cm (cm^2^ s^−1^ cmHg)) was obtained from the average value of more than three experiments using the following equation:4
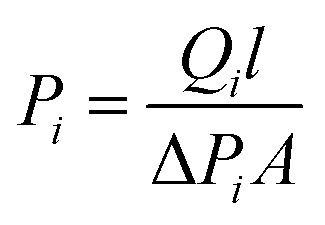
Where *Q*_*i*_, *l*, Δ*P*_*i*_ and A are the gas volumetric flow rate of the gas (cm^3^ s^−1^) (STP), membrane thickness (cm), transmembrane pressure difference (cmHg) and effective membrane area (cm^2^), respectively. The pure gas ideal selectivity (*α*_*ij*_) was obtained using eqn (5):5
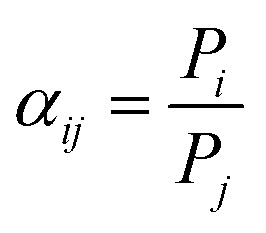


The permeability of the dry membrane is given by eqn (6):

6 *P*_*i*_ = *D*_*i*_ × *S*_*i*_

where *D*_*i*_ and *S*_*i*_ are the diffusion (cm^2^ s^−1^) and solubility coefficients (cm^3^ (STP) cm^−3^ cmHg^−1^) of gas ‘*i*’ in the polymer membrane, respectively. The diffusion coefficients *D*_*i*_ were measured by the time lag method^14,41,42^ using eqn (7):7
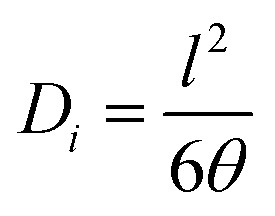
Where *θ* is the diffusivity time lag and *l* is the thickness of the membrane. The solubility coefficient *S*_*i*_ was obtained using eqn (6).

The membrane samples were dried under a vacuum for 24 h prior to testing. In this study, N_2_/CO_2_ was used as the feed gas. The pressure and temperature of the high-pressure side were maintained at 1 bar and 30 °C, respectively. For each membrane, the gas permeation was tested three times to ensure that the error range of the gas permeability was within 5% and that for the gas selectivity was within 8%. The errors of the gas diffusivity coefficient and solubility coefficient of dry membranes were all less than 10%.

## 3 Results and discussion

### 3.1 Characterization of nanofillers

**Fig. 1:**
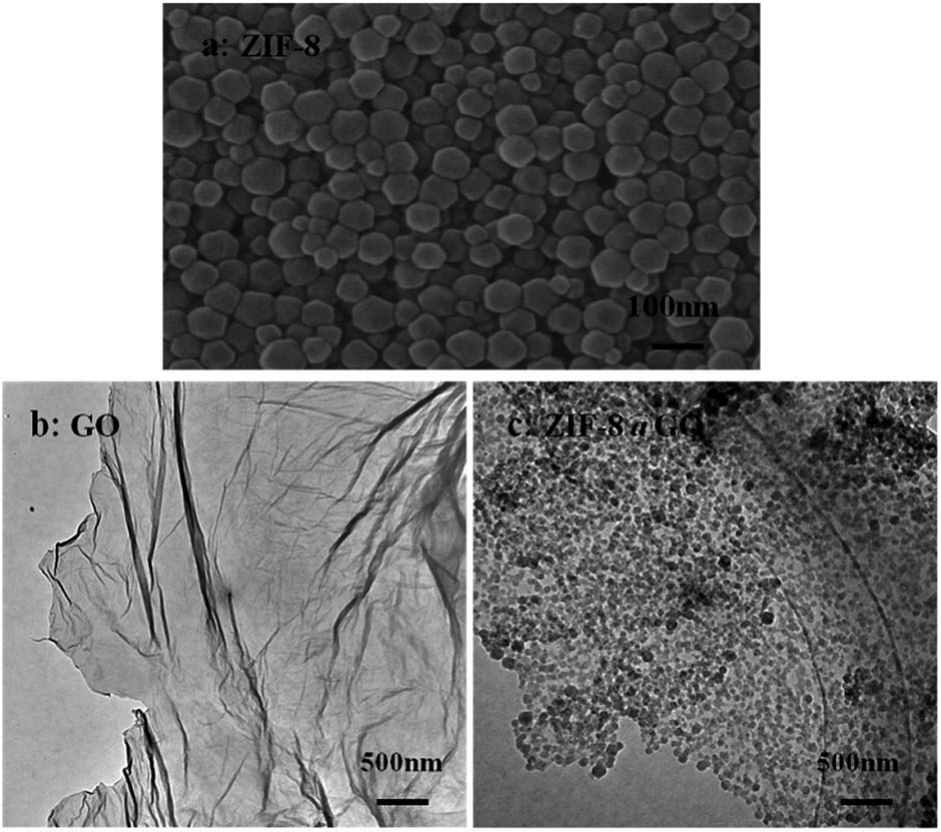
SEM image of (a) ZIF-8 and TEM image of (b) GO and (c) ZIF-8@GO.

The size and morphology of GO, ZIF-8, and ZIF-8@GO were observed using SEM and TEM. The ZIF-8 nanoparticles are a rhombic dodecahedron shape with sizes in the range of 50–60 nm as shown by SEM (Fig. 1a). GO is fully exfoliated into ultrathin nanosheets as shown by TEM (Fig. 1b). The size and morphology of ZIF-8@GO are similar to pristine GO; the difference is that ZIF-8 was grown *in situ* on the surface of GO. The TEM image (Fig. 1c) demonstrates a homogeneous distribution of ZIF-8 on GO. In addition, the ZIF-8 does not show any visible aggregation.

**Fig. 2:**
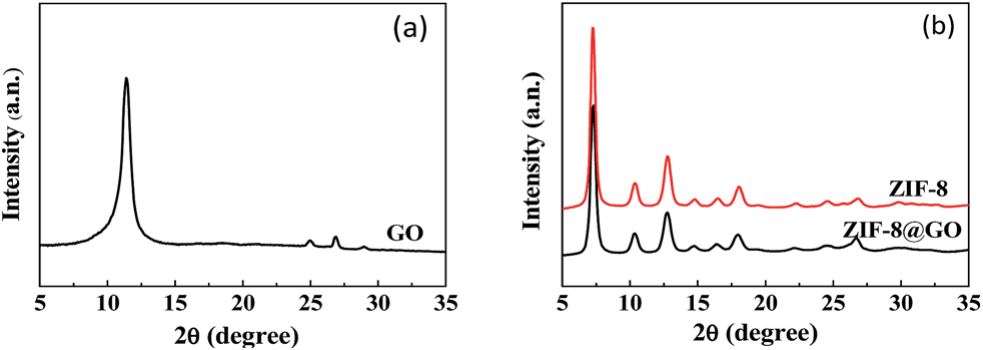
XRD patterns recorded for (a) GO and (b) ZIF-8, ZIF-8@GO.

The XRD patterns of GO, ZIF-8 and ZIF-8@GO are shown in Fig. 2. The XRD pattern of the GO nanosheets has a strong peak at 2*θ* = 11.6°. The distance between the corresponding chain (*d*-spacing) is 0.765 nm, indicating that GO was successfully exfoliated into single layer ultrathin nanosheets.^43^ However, the strong diffraction peak of GO in ZIF-8@GO disappears; the reason is that the content of GO in ZIF-8@GO was too low to be examined. The pattern of ZIF-8@GO is similar to pristine ZIF-8 with another diffraction peak exhibited at about 8°.^36^

**Fig. 3:**
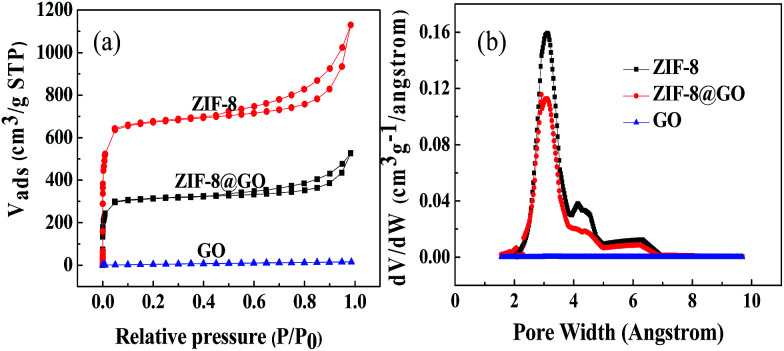
(a) Nitrogen adsorption–desorption isotherms and (b) pore size distribution curves observed for ZIF-8, ZIF-8@GO and GO.

Fig. 3(a) shows the N_2_ adsorption–desorption isotherms at 77 K observed for ZIF-8, ZIF-8@GO and GO. The specific surface area decreases from 1964 m^2^ g^−1^ for ZIF-8 to 1413 m^2^ g^−1^ for ZIF-8@GO. This indicates that GO occupies a certain amount of the pores in ZIF-8. The pore size distribution of ZIF-8, ZIF-8@GO and GO is shown in Fig. 3(b). The pore size distribution of ZIF-8@GO is similar to ZIF-8 at 2–4 nm.

**Fig. 4:**
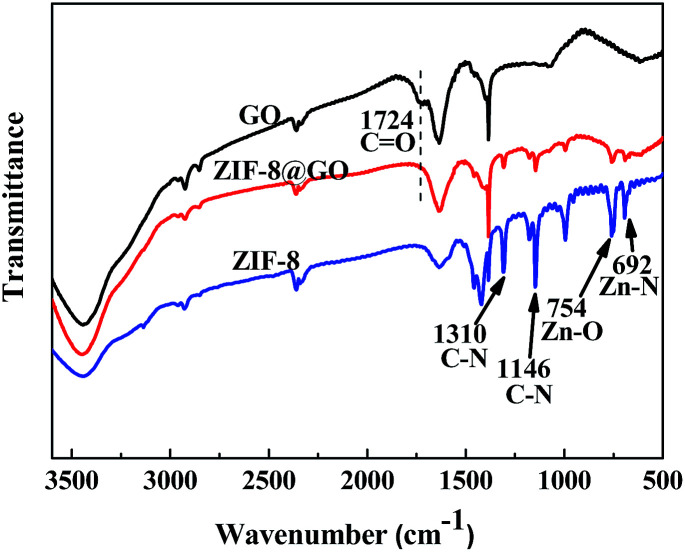
FT-IR spectra recorded for GO, ZIF-8 and ZIF-8@GO.

When compared with the GO nanosheets, the FT-IR spectra of ZIF-8@GO does not have a peak at 1724 cm^−1^, corresponding to C=O, as shown in Fig. 4.^44^ Other bands at 1146 cm^−1^ and 1310 cm^−1^, corresponding to the C–N bonds in the imidazole group, 754 cm^−1^, corresponding to the Zn–O bonds, and 692 cm^−1^, corresponding to Zn–N bonds, were ascribed to the ZIF-8 structure.^38,45^

**Fig. 5:**
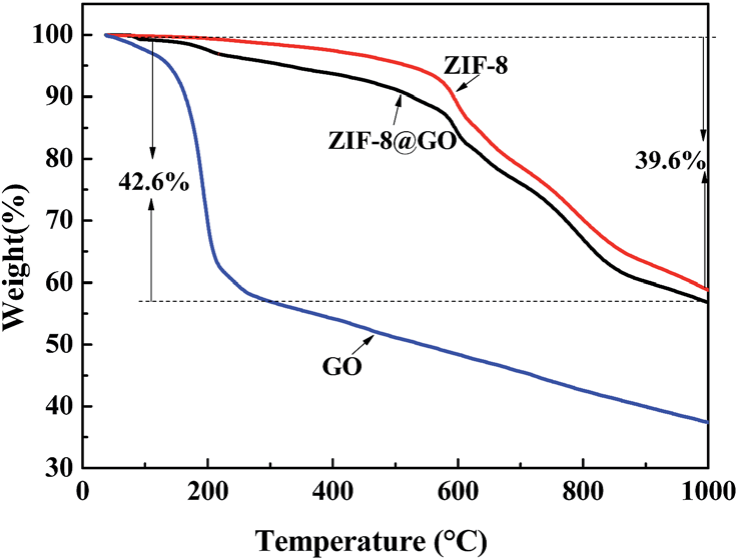
TGA curves observed for GO, ZIF-8 and ZIF-8@GO.

TGA was performed to analyze the thermal stability of the fillers and the ratio of GO and ZIF-8 in ZIF-8@GO was estimated (Fig. 5). The weight loss of ZIF-8@GO at 150–200 °C is attributed to the thermal decomposition of GO and the weight loss starting from 200 °C is attributed to the thermal decomposition of ZIF-8. Based on the obtained data, the content of GO and ZIF-8 in ZIF-8@GO was about 5% and 95%, respectively.

### 3.2 Characterization of membranes

**Fig. 6:**
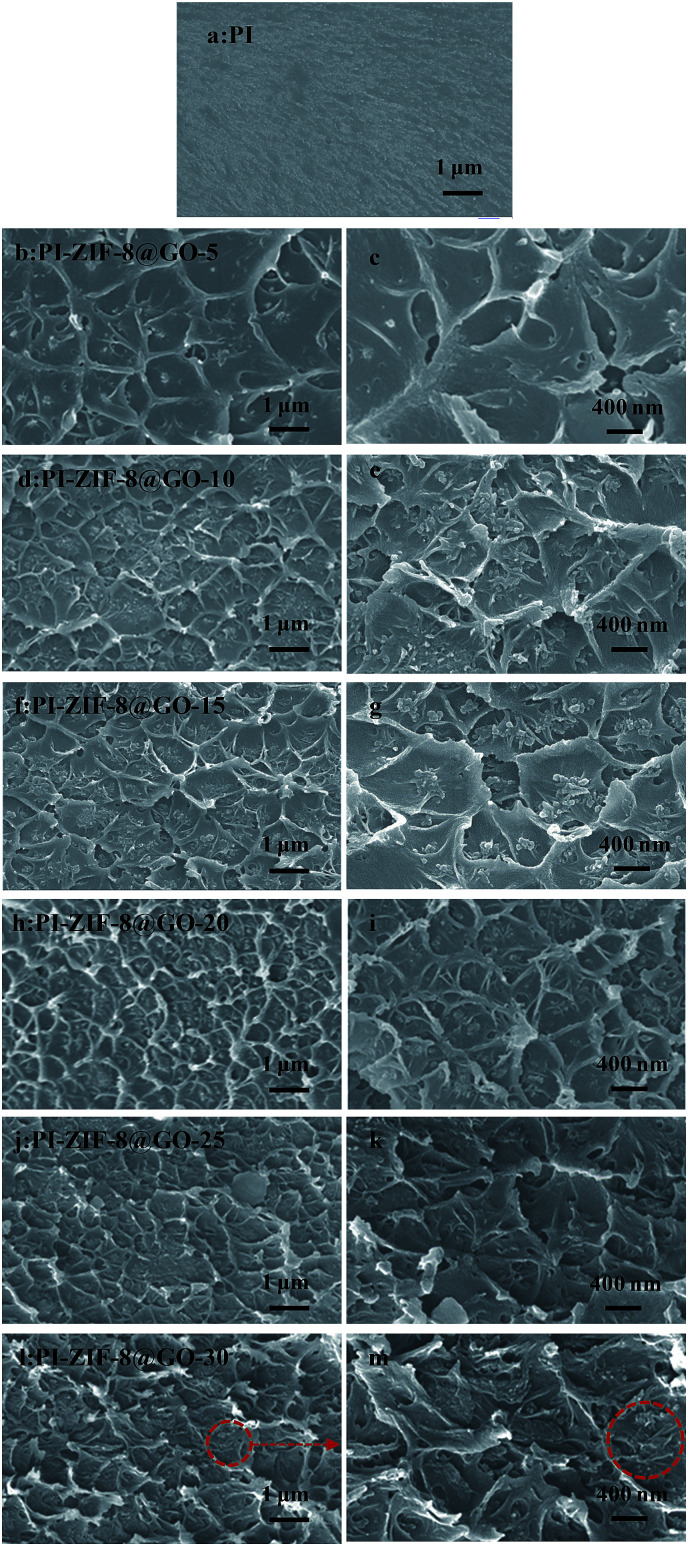
Cross-section FESEM images of (a) unfilled PI, (b, c) PI-ZIF-8@GO-5, (d, e) PI-ZIF-8@GO-10, (f, g) PI-ZIF-8@GO-15, (h, i) PI-ZIF-8@GO-20, (j, k) PI-ZIF-8@GO-25 and (l, m) PI-ZIF-8@GO-30.

The cross-sectional morphologies of the membranes were characterized by FESEM as shown in Fig. 6. The membrane structures were strongly influenced by the incorporation of the fillers. When compared to the unfilled PI membrane (Fig. 6a) with a smooth and dense morphology, the MMMs show a rougher cross-section. Fig. 6b–i reveals that at low ZIF-8@GO loadings, the fillers are dispersed homogeneously in the PI matrix, resulting in a relatively uniform cross-sectional structure. The cross-sectional image of PI-ZIF-8@GO-20 shows that ZIF-8@GO was well-dispersed in the PI matrix, implying the good compatibility between ZIF-8@GO and the PI matrix. As the ZIF-8@GO content increases, *e.g.*, PI-ZIF-8@GO-25 and PI-ZIF-8@GO-30 membranes (Fig. 6j–m), ZIF-8@GO tends to slightly aggregate in the membrane.

**Fig. 7:**
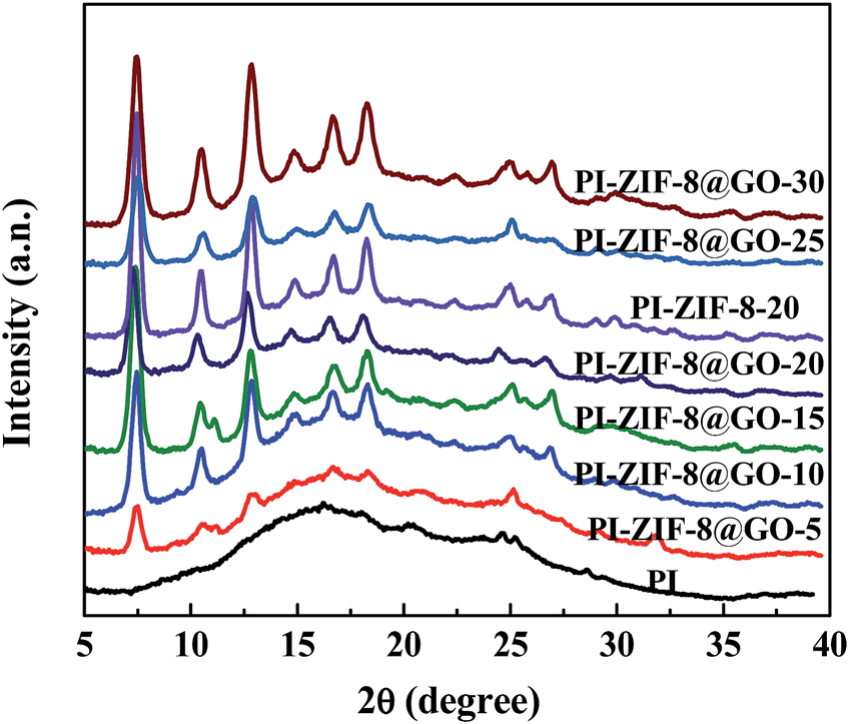
XRD patterns recorded for the membranes.

The XRD spectra of the unfilled PI and the MMMs with different filler content are presented in Fig. 7. The unfilled PI membrane shows broad and strong peaks at 10–30°, which result from the crystalline region of the polyamide segment.^46,47^ However, the MMMs have both the broad and characteristic peaks of the fillers, which imply that the crystallinity of the fillers was not affected by the PI matrix.

**Fig. 8:**
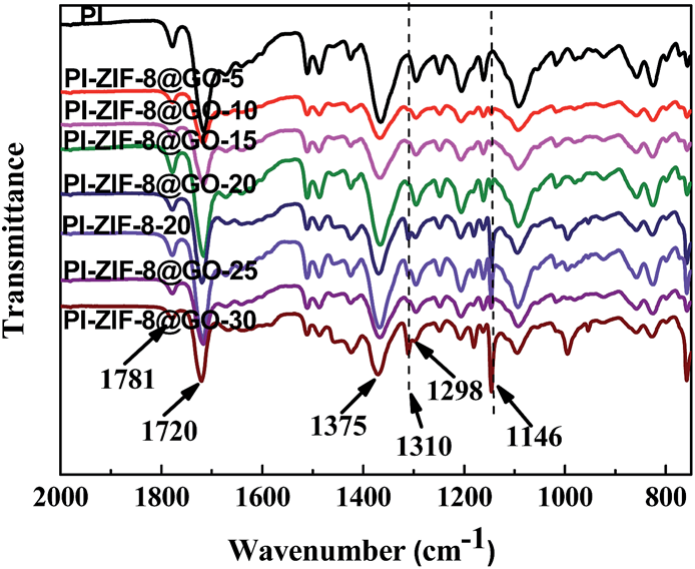
FT-IR spectra recorded for the membranes.

The FT-IR spectra of the unfilled PI membrane and ZIF-8@GO loaded MMMs are presented in Fig. 8. The characteristic peaks at 1781 cm^−1^ and 1720 cm^−1^ correspond to the C=O bond stretching vibrations of the imide groups and 1375 cm^−1^ was attributed to the C–N stretching vibrations of the imide group for the unfilled PI membrane.^48^ The peak at 1298 cm^−1^ was attributed to the bending vibrations of the C–CO–C groups.^49^ The FT-IR spectra observed for the MMMs are similar to the unfilled PI membrane with no significant change. However, upon the incorporation of ZIF-8 or ZIF-8@GO, the two new peaks at 1146 cm^−1^ and 1310 cm^−1^ were attributed to the C–N stretching vibrations in the imidazole groups, which proves that the ZIF-8 or ZIF-8@GO are well incorporated into the polymer matrix and retains the original chemical structure.

**Fig. 9:**
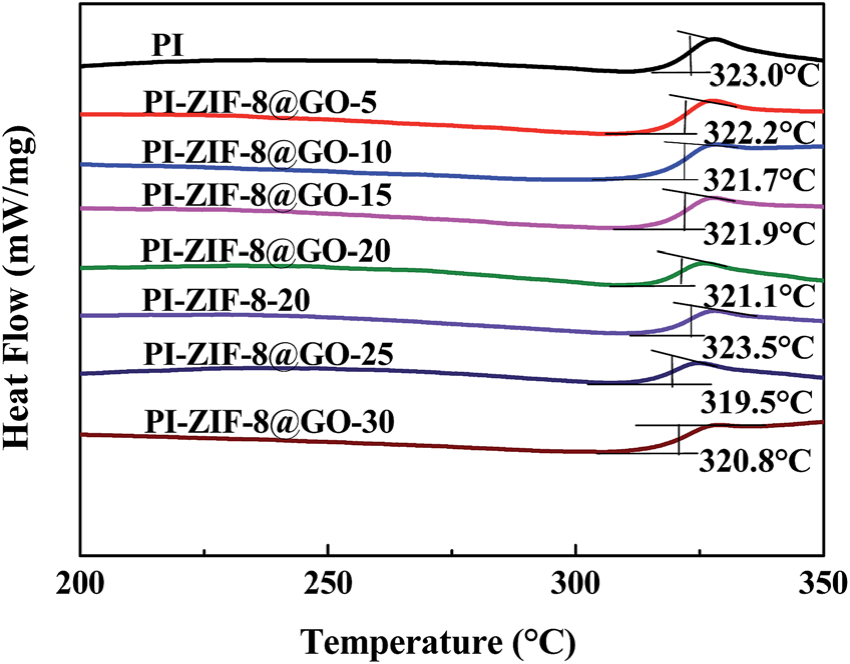
DSC curves obtained for the membranes.

The glass transition temperature (*T*_g_) of the membranes were detected using DSC. The unfilled PI membrane exhibits a *T*_g_ at 323.0 °C as shown in Fig. 9. The *T*_g_ of all the MMMs, except for the ZIF-8 filled membrane, shows a slight decrease when compared with the unfilled PI membrane. The *T*_g_ of the ZIF-8@GO filled membranes (from 323.0 to 320.8 °C) gradually decreases as the ZIF-8@GO content increases. The decline in *T*_g_ indicates that the incorporation of the fillers increases the chain mobility of PI. In general, the incorporation of GO leads to the rigidity of the polymer chain.^19^ In this study, the membranes do not show evident rigidity because the growth of ZIF-8 on the GO interferes with the interaction between GO and PI. Furthermore, the *T*_g_ of the PI-ZIF-8-20 filled membrane (323.5 °C) is higher than all the ZIF-8@GO filled membranes and unfilled PI membrane because the high surface area of the ZIF-8 nanoparticles increases the contact area between the polymer and fillers, thus increasing the interactions that inhibit the chain mobility of PI.

**Fig. 10:**
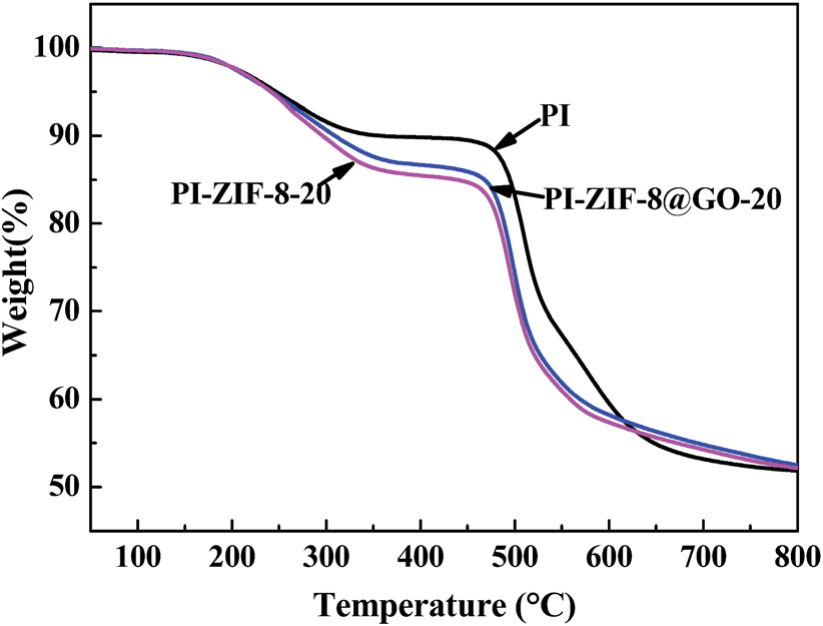
TGA curves obtained for the unfilled PI, PI-ZIF-8@GO-20 and PI-ZIF-8-20 membranes.

The thermal stability of the membranes was analyzed using TGA as shown in Fig. 10. The three typical membranes, which are unfilled PI, PI-ZIF-8@GO-20 and PI-ZIF-8-20 were tested. The TGA curves of the membranes have two main degradation processes: the first phase of weight loss at 240–350 °C resulted from of the decomposition of the organic ligands in ZIF-8; the second stage of weight loss at ∼450 °C is primarily ascribed to the PI chain decomposition. Before 625 °C, the thermal stability was as follows: PI > PI-ZIF-8@GO-20 > PI-ZIF-8-20. Above 625 °C, the thermal stability was in the order: PI-ZIF-8@GO-20 > PI-ZIF-8-20 > PI. Moreover, the decomposition rate of PI-ZIF-8@GO-20 is slightly slower than that of PI-ZIF-8-20 throughout the TGA analysis.

### 3.3 Water uptake and water state

The content of free water in the MMMs is higher than that of the unfilled PI membrane and exhibits a maximum value with 43.52% at a ZIF@GO loading of 30 wt% as shown in Table 1. Moreover, the content of bound water in the MMMs is higher than that of unfilled PI membrane, but reaches a maximum value when the ZIF@GO loading is 20 wt%.

**Table 1:** Water uptake and water state of unfilled Matrimid® 5218 membrane and MMMs

Sample	Total water (*W*_t_, %)	Free water (*W*_f_, %)	Bound water (*W*_b_, %)
PI	3.30	2.79	0.51
PI-ZIF-8@GO-5	8.66	7.46	1.20
PI-ZIF-8@GO-10	12.90	12.02	0.88
PI-ZIF-8@GO-15	18.72	17.81	0.91
PI-ZIF-8@GO-20	32.00	30.25	1.75
PI-ZIF-8@GO-25	29.30	27.79	1.51
PI-ZIF-8@GO-30	43.52	42.61	0.92
PI-ZIF-8-20	21.35	18.94	2.41
PI-GO-20	4.21	3.33	0.88

### 3.4 Gas separation performance of the membranes

The pure gas permeability and ideal selectivity of the dry and humidified membranes were investigated (Table 2). To further investigate the gas transport mechanism, the diffusion coefficient (*D*) and the solubility coefficient (*S*) of CO_2_ and N_2_ for the dry membranes and their corresponding diffusion selectivity and solubility selectivity are determined and listed in Table 3. As expected, the diffusion coefficient of gas increases for the MMMs when compared with the unfilled PI membrane (Table 3). The CO_2_ diffusion coefficient increases from 2.75 × 10^8^ cm^2^ s^−1^ for the unfilled PI membrane to 5.41 × 10^8^ cm^2^ s^−1^ for the PI-ZIF-8@GO-20 membrane. This increase in the diffusion coefficient is primarily attributed to the synergistic effect of the modestly improved chain mobility, as shown by DSC results, and the increased transport pathways with sizes of 0.34 nm in ZIF-8. Similar to the diffusion coefficient, the MMMs show an enhanced CO_2_ solubility coefficient when compared with the unfilled PI membrane. The MMMs contain ZIF-8, which shows CO_2_ affinity, and provide ether-oxygen groups from GO for the CO_2_ molecules. Moreover, the PI-ZIF-8@GO-20 membrane shows a higher diffusion selectivity and solubility selectivity than the other MMMs for CO_2_/N_2_ gas. The membrane loaded with ZIF-8@GO at a loading of 20 wt% shows an increased CO_2_/N_2_ diffusion selectivity and solubility selectivity by 12% and 44%, respectively, when compared with the unfilled PI membrane.

**Table 2:** Pure gas permeability and ideal CO_2_/N_2_ selectivity of the dry membranes and humidified membranes

Sample	Dry membranes			Humidified membranes		
	*P*_CO_2__	*P*_N_2__	*α*_CO_2_/N_2__	*P*_CO_2__	*P*_N_2__	*α*_CO_2_/N_2__
PI	6.62	0.20	33.10	52	1.44	36
PI-ZIF-8@GO-5	9.28	0.28	33.14	—	—	—
PI-ZIF-8@GO-10	7.32	0.18	40.67	84	1.82	46
PI-ZIF-8@GO-15	14.50	0.31	46.77	124	2.51	49
PI-ZIF-8@GO-20	11.14	0.21	53.05	238	3.65	65
PI-ZIF-8@GO-25	14.32	0.29	49.40	—	—	—
PI-ZIF-8@GO-30	21.80	0.64	34.06	259	6.59	39
PI-ZIF-8-20	12.31	0.30	41.03	178	4.23	42
PI-GO-20	8.23	0.23	35.78	134	3.70	36

**Table 3:** Gas diffusivity coefficient and solubility coefficient of the dry membranes loaded with GO, ZIF-8 and ZIF@GO, respectively (1 bar, 30 °C)

Membrane	*D* (× 10^−8^ cm^2^ s^−1^)		*S* (× 10^−2^ cm^3^ (STP)/(cm^3^ cmHg))		*D*_CO_2__/*D*_N_2__	*S*_CO_2__/*S*_N_2__
	CO_2_	N_2_	CO_2_	N_2_		
PI	2.75	1.46	2.41	0.14	1.88	17.57
PI-ZIF-8@GO-5	3.18	1.69	2.92	0.17	1.88	17.61
PI-ZIF-8@GO-10	2.93	1.51	2.50	0.12	1.94	20.96
PI-ZIF-8@GO-15	4.07	2.08	3.56	0.15	1.96	23.90
PI-ZIF-8@GO-20	3.51	1.67	3.17	0.13	2.10	25.24
PI-ZIF-8@GO-25	4.02	2.03	3.56	0.14	1.98	23.94
PI-ZIF-8@GO-30	5.41	2.96	4.03	0.22	1.83	18.64
PI-ZIF-8-20	3.71	1.83	3.32	0.16	2.03	20.24
PI-GO-20	3.01	1.57	2.73	0.15	1.92	18.66

Both the CO_2_ permeability and the selectivity of all the humidified membranes were significantly improved when compared with the CO_2_ permeability and selectivity of all the dry membranes (Table 2). For the unfilled PI membrane in its dry state, the CO_2_ permeability was 6.6 barrer, which increased to 52 barrer in its humidified state, thus increasing by 685%. Water plays an important role in gas transport for the humidified PI membrane. Water may swell and plasticize the PI polymer matrix, strengthening the intersegmental mobility of the polymer chains and enhance the gas diffusivity. Moreover, water may produce additional transport channels for gas transport. Consequently, the positive influence of water leads to the enhanced gas permeability. For the humidified MMMs, the CO_2_ permeability increases upon increasing the ZIF-8@GO content. When compared with the unfilled PI membrane, the CO_2_ permeability and CO_2_/N_2_ selectivity of the PI-ZIF-8@GO-20 membrane increase by 358% and 81%, respectively. The introduction of ZIF-8@GO improves the water content in the MMMs, which increases the dissolved CO_2_ amount and simultaneously constructs interconnected CO_2_ transport pathways in the MMMs, thus enhancing the CO_2_ permeability and selectivity.

**Fig. 11:**
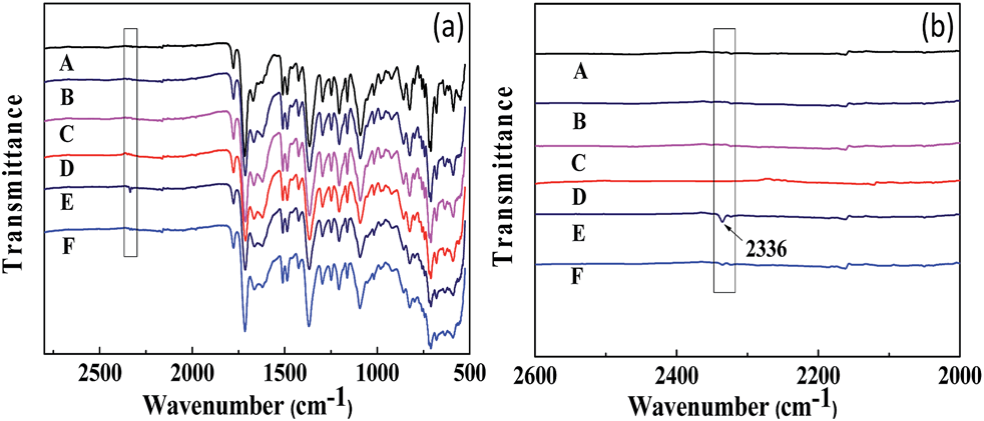
FT-IR spectra of CO_2_ adsorption and desorption within the humidified membranes. (A) PI membrane; (B) CO_2_-absorbed PI membrane; (C) CO_2_-desorbed PI membrane; (D) PI-ZIF-8@GO-20 membrane; (E) CO_2_-absorbed PI-ZIF-8@GO-20 membrane; (F) CO_2_-desorbed PI-ZIF-8@GO-20.

The FT-IR spectra obtained for CO_2_ adsorption and desorption are shown in Fig. 11. All the membranes do not show any significant change in the FT-IR spectra after humidification, adsorption and desorption, while the CO_2_-absorbed PI-ZIF-8@GO-20 membrane in its humidified state shows a new infrared absorption peak at 2336 cm^−1^, which was assigned to the adsorption band of water–CO_2_, indicating the CO_2_ adsorption in the membranes. The peak at 2336 cm^−1^ disappears in the CO_2_-desorbed PI-ZIF-8@GO-20 membrane, indicating that the reversible interaction disappears, while only physical adsorption still exists in the membrane. However, there is no corresponding peak in the unfilled membrane. There is probably less water in the unfilled membrane, resulting in less CO_2_ adsorption. In short, water effectively facilitates the transport of CO_2_ in the humidified MMMs.

In the humidified state, for the PI-ZIF-8@GO MMMs, the CO_2_ permeability and CO_2_/N_2_ selectivity increase as the loading of ZIF-8@GO increases up to 20 wt%, indicating the absence of non-selective defects. However, when the loading of ZIF-8@GO was 30 wt%, the significantly increased permeability and reduced selectivity were ascribed to the visible aggregation of ZIF-8@GO in the MMMs as shown by SEM. The CO_2_ permeability increases from 52 barrer for the unfilled PI to 259 barrer for the PI-ZIF-8@GO loaded MMMs at 30 wt% loading. The ideal CO_2_/N_2_ selectivity increases from 36 for the unfilled PI membrane to 65 for the PI-ZIF-8@GO loaded MMMs at 20 wt% loading. The increased CO_2_ permeability results from the following reasons. First, the content of free water in the membranes increases when compared with the unfilled PI membrane as listed in Table 1. The water swells the PI matrix and produces more CO_2_ transport passageways in the MMMs, resulting in the increased CO_2_ permeability. Second, the increased CO_2_ transport channels in ZIF-8 with sizes of 0.34 nm and additional CO_2_ transport channels at the ZIF-8-GO interface lead to an increase in the CO_2_ permeability. The MMM with 20 wt% ZIF-8@GO exhibits the optimum gas separation performance with a CO_2_ permeability of 238 barrer and CO_2_/N_2_ selectivity of 65, which is 458% and 180% higher than the pure membrane, respectively, thus surpassing the 2008 Robeson upper boundary line. The gas separation performance of PI-ZIF-8@GO-20 surpasses or is close to the gas separation as reported (Table 4).^38^

**Table 4:** Comparison of the gas permeability and selectivity of previously reported Matrimid-based MMMs with that of the MMMs determined in this study

Filler	Loading (wt%)	Polymer	Operating conditions				*P*_CO_2__ [barrer]	*P*_CO_2__/*P*_N_2__	Ref.
			Test state	Analysis	*T* (°C)	Δ*P* (bar)			
MIL-101	10	Matrimid®5218	dry state	Single gas	35	10	6.95	52.92	50
ZIF-90	15	6FDA-DAM	dry state	Single gas	25	2	720	22	51
MIL-53	37.5	Matrimid®5218	dry state	Single gas	35	2	51.0	28.3	52
CU-BPY-HFS	30	Matrimid®5218	dry state	Single gas	35	2.0	10.4	33.5	53
MOF-5	30	Matrimid®5218	dry state	Single gas	35	2	20.2	39	30
ZIF-8	10	Matrimid®5218	dry state	Single gas	22	4	13.67	21.6	54
Mesoporous silica	8	Matrimid®5218	dry state	Mixture	25	1.75	15.3	40.3	55
UiO-66-NH_2_	23	Matrimid®5218	dry state	Single gas	25	1.36	23.7	36.5	56
PA^a^-UiO-66-NH_2_	23	Matrimid®5218	dry state	Single gas	25	1.38	29	37	56
SO_3_H-MCM-41	30	Matrimid®9725	dry state	Mixture	25	10	9.4	31.5	57
SO_3_H-MCM-41	30	Matrimid®9725	dry state	Single gas	25	10	10.4	37.4	57
PEGSS	20	Matrimid®5218	dry state	Single gas	30	1	8.21	61.24	58
CSM-23.3	30	Matrimid®9725	dry state	Mixture	35	9	52.6	37.6	59
POP-2	20	Matrimid®5218	dry state	Single gas	35	2	25	25	60
Cu-BTC	30	Matrimid®5218	dry state	Single gas	35	2	54	28.5	60
ZIF-8	30	Matrimid®5218	dry state	Single gas	35	2	40.1	24.5	60
MIL-125	15	Matrimid®9725	dry state	Mixture	35	9	9.4	34	61
NH_2_-MIL-125	15	Matrimid®9725	dry state	Mixture	35	9	9.1	38	61
Mg_2_(dobdc)	10	6FDA/TMPDA	dry state	Single gas	25	2	850	23	62
Cd–6F	10	6FDA-ODA	dry state	Single gas	25	2	37.8	35.1	63
[Cu_3_(BTC)_2_]	30	Matrimid®9725	dry state	Mixture	35	10	18.8^b^	24.1	64
ZIF-8	30	Matrimid®9725	dry state	Mixture	35	10	19.7^b^	19.5	64
MIL-53(Al)	30	Matrimid®9725	dry state	Mixture	35	10	19.3^b^	23.6	64
ZIF-8	20	Matrimid® 5218	dry state	Single gas	30	1	12.31	41.03	This study
GO	20	Matrimid® 5218	dry state	Single gas	30	1	8.23	35.78	This study
ZIF-8@GO	20	Matrimid® 5218	dry state	Single gas	30	1	11.14	53.05	This study
ZIF-8	20	Matrimid® 5218	Humidified state	Single gas	30	1	178	42.12	This study
GO	20	Matrimid® 5218	Humidified state	Single gas	30	1	134	36.18	This study
ZIF-8@GO	20	Matrimid® 5218	Humidified state	Single gas	30	1	238	65.23	This study

a PA = phenyl acetyl group.

b PCO_2_ units GPU.

When compared to the unfilled PI membrane, PI-ZIF-8@GO MMMs show a higher CO_2_/N_2_ selectivity. The ZIF-8 with high surface area in the PI-ZIF-8@GO MMMs may effectively enhance the adsorption capacity towards CO_2_, resulting in the increased solubility selectivity. Moreover, when compared to the unfilled PI membrane, more free water exists in the PI-ZIF-8@GO loaded MMMs, which leads to the relatively lower transport resistance of CO_2_ than that of N_2_ with high CO_2_/N_2_ selectivity. In addition, the increased CO_2_/N_2_ diffusion selectivity causes the enhanced CO_2_/N_2_ selectivity. In comparison, the ZIF-8@GO are more effective in facilitating CO_2_ transport than that of single ZIF-8 or GO in the MMMs. The underlying reason is that the ZIF-8@GO with uniform pore sizes of 0.34 nm, additional CO_2_ transport channels at the interface of ZIF-8 and GO, and oxygen-containing functional groups on GO as well as the good interface compatibility between PI matrix and ZIF-8@GO constructs high-performance CO_2_ transport pathways in the MMMs.

### 3.5 Mixed gas separation performance

**Fig. 12:**
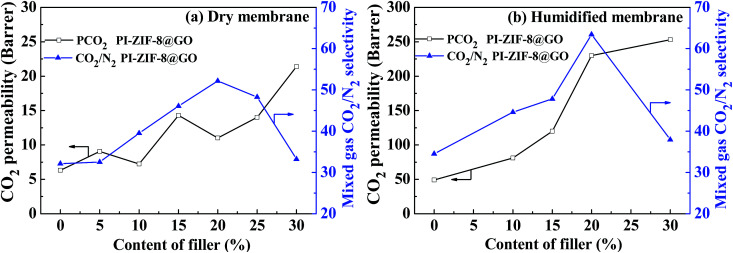
Comparison of the mixed gas separation performance observed for the dry and humidified membranes: (a) CO_2_ permeability and CO_2_/N_2_ selectivity observed for the dry membranes; (b) CO_2_ permeability and CO_2_/N_2_ selectivity observed for the dry membranes with the ZIF-8@GO based MMMs (1.0 bar, 30 °C).

Fig. 12 shows the separation performance of the unfilled PI membrane and the MMMs in a mixed gas. For the unfilled PI membrane, the mixed gas-real selectivity was lower than the corresponding ideal selectivity value of pure gas. However, the PI-ZIF-8@GO loaded MMMs exhibit real selectivity similar to their ideal value, suggesting the negligible competitive adsorption between CO_2_ and N_2_ in the MMMs. Since in water the solubility of CO_2_ is remarkably higher than that of N_2_ and PI-ZIF-8@GO loaded MMMs hold more water, the CO_2_ transport pathways are multiplied with no evident competitive adsorption caused by N_2_.

### 3.6 The effect of operating pressure

**Fig. 13:**
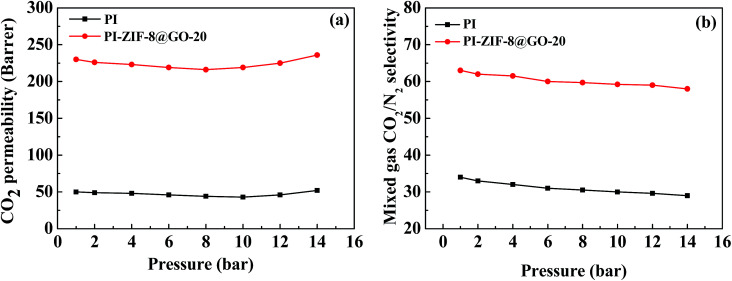
Effect of feed pressure on (a) CO_2_ permeability and (b) CO_2_/N_2_ selectivity of humidified membranes.

The effect of operating pressure was investigated in the range of 2–14 bar as shown in Fig. 13. The CO_2_ permeability exhibits minor dependence on the gas pressure. The CO_2_/N_2_ selectivity reduces as the feed pressure increases. When the pressure is lower than 8 bar, the CO_2_ permeability decreases with an increase in the pressure, resulting from the saturation of the Langmuir absorption sites. At pressures up to 14 bar, the high concentration of CO_2_ swells the PI chains and strengthens the mobility of the chain, leading to the increased CO_2_ permeability. Moreover, N_2_ transport is enhanced due to the enhanced mobility of the polymer chains and increased free volume in the membranes, leading to a reduced selectivity. Consequently, the plasticization phenomenon is not severe, which is primarily ascribed to the presence of water as a plasticizer in the PI matrix.

### 3.7 Long-term operation stability

**Fig. 14:**
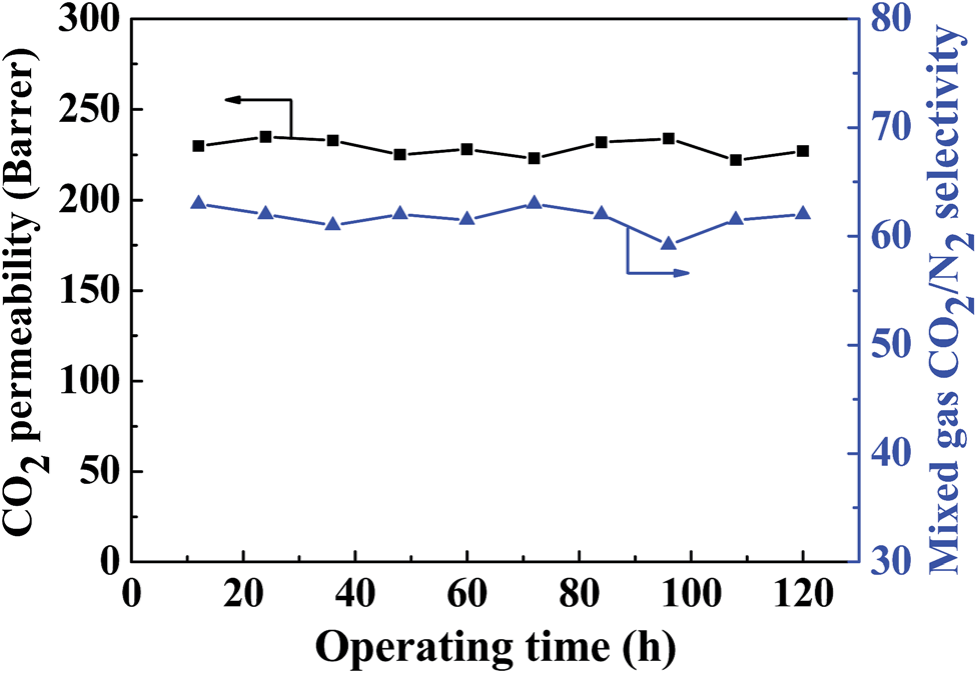
Long-term operation stability of the gas separation performance observed for the MMM containing 20 wt% ZIF-8@GO.

As shown in Fig. 14, the long-term gas separation performance of the MMM containing 20 wt% ZIF-8@GO was investigated for up to 120 h. The CO_2_ permeability and CO_2_/N_2_ selectivity fluctuate within a narrow range during this test. The MMM containing 20 wt% ZIF-8@GO exhibits favorable operation stability, indicating the structural stability of the MMM for potential application in gas separation.

### 3.8 Comparison of the CO_2_/N_2_ separation performance with Robeson's upper boundary

**Fig. 15:**
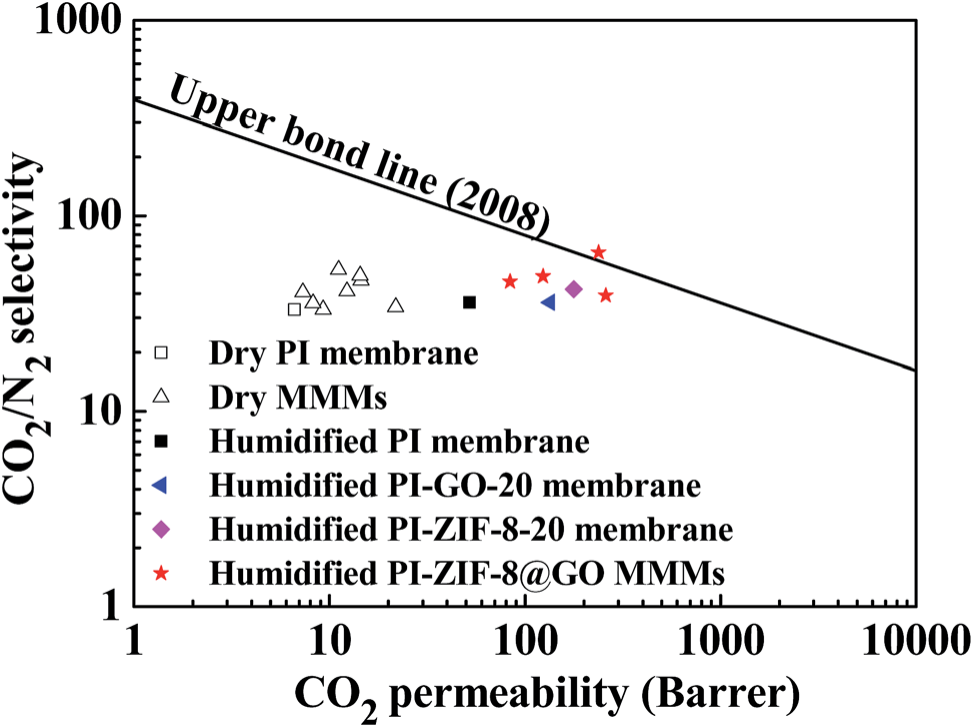
Robeson's plots for CO_2_/N_2_ separation.

Fig. 15 shows a comparison of the CO_2_/N_2_ separation performance with Robeson's upper boundary. In the humidified MMMs, the gas separation performance is close to or surpasses the Robeson's upper boundary reported in 2008, while in the dry MMMs, the gas separation performance falls far below the upper boundary. Both the CO_2_ permeability and the CO_2_/N_2_ selectivity are remarkably improved in the PI-ZIF-8@GO MMMs, confirming the benefits of the synergistic effect of ZIF-8 and GO in the MMMs towards enhancing the CO_2_ separation performance.

## 4 Conclusions

ZIF-8@GO was prepared using a facile *in situ* growth method and MMMs comprising PI and ZIF@GO were fabricated. The gas separation performance of the membranes was investigated and the CO_2_ permeability and CO_2_/N_2_ selectivity of the ZIF-8@GO loaded MMMs increased when compared with that of the unfilled PI membrane. In particular, the membrane containing ZIF-8@GO exhibits the highest selectivity of up to 65 for the CO_2_/N_2_ system with a CO_2_ permeability of 238 barrer, which surpasses the Robeson's upper boundary reported in 2008. The MMMs containing ZIF-8@GO show remarkable increments in the CO_2_/N_2_ selectivity when compared with the MMMs containing single ZIF-8 or GO fillers at the same content. The ZIF-8@GO loaded MMMs with high CO_2_ separation performance are attributed to the ZIF-8@GO nanocomposite materials combining the favorable advantages of GO and ZIF-8. First, the high-aspect ratio of the GO nanosheets enhanced the diffusivity selectivity and ZIF-8 with high porosity is beneficial to the improvement of the CO_2_ permeability. Second, ZIF-8 with high porosity is beneficial to the improvement of the CO_2_ permeability. Third, ZIF-8@GO may construct extra CO_2_ transport channels at the interface of ZIF-8 and GO. In their humidified state, the improved permeability is primarily ascribed to the incremental amount of free water, which produces more CO_2_ transport passageways in the MMMs and the elevated content of bound water as well as the good interface compatibility between ZIF-8@GO and the PI matrix.

## Conflicts of interest

There are no conflicts to declare.

## Supplementary Material
